# Triatomine Feeding Profiles and *Trypanosoma cruzi* Infection, Implications in Domestic and Sylvatic Transmission Cycles in Ecuador

**DOI:** 10.3390/pathogens10010042

**Published:** 2021-01-07

**Authors:** Sofía Ocaña-Mayorga, Juan José Bustillos, Anita G. Villacís, C. Miguel Pinto, Simone Frédérique Brenière, Mario J. Grijalva

**Affiliations:** 1 Centro de Investigación para la Salud en América Latina (CISeAL), Escuela de Ciencias Biológicas, Facultad de Ciencias Exactas y Naturales, Pontificia Universidad Católica del Ecuador, Calle San Pedro y Pamba Hacienda, Nayón 170530, Ecuador; sbocana@puce.edu.ec (S.O.-M.); juanjobustillos4@gmail.com (J.J.B.); agvillacis@puce.edu.ec (A.G.V.); frederique.breniere@ird.fr (S.F.B.); 2 Department of Biomedical Sciences, Infectious and Tropical Disease Institute, Heritage College of Osteopathic Medicine, Ohio University, Athens, OH 45701, USA; 3 Observatorio de Biodiversidad Ambiente y Salud (OBBAS), Quito 170517, Ecuador; pinto@obbas.org; 4 Intertryp, IRD-Cirad, TA A-17/G Campus International de Baillarguet, Université de Montpellier, CEDEX 5, 34398 Montpellier, France

**Keywords:** Chagas disease, *Trypanosoma cruzi*, triatomines, blood meal source, Ecuador

## Abstract

Understanding the blood meal patterns of insects that are vectors of diseases is fundamental in unveiling transmission dynamics and developing strategies to impede or decrease human–vector contact. Chagas disease has a complex transmission cycle that implies interactions between vectors, parasites and vertebrate hosts. In Ecuador, limited data on human infection are available; however, the presence of active transmission in endemic areas has been demonstrated. The aim of this study was to determine the diversity of hosts that serve as sources of blood for triatomines in domestic, peridomestic and sylvatic transmission cycles, in two endemic areas of Ecuador (central coastal and southern highland regions). Using conserved primers and DNA extracted from 507 intestinal content samples from five species of triatomines (60 *Panstrongylus chinai*, 17 *Panstrongylus howardi*, 1 *Panstrongylus rufotuberculatus*, 427 *Rhodnius ecuadoriensis* and 2 *Triatoma carrioni*) collected from 2006 to 2013, we amplified fragments of the *cytb* mitochondrial gene. After sequencing, blood meal sources were identified in 416 individuals (146 from central coastal and 270 from southern highland regions), achieving ≥ 95% identity with GenBank sequences (NCBI-BLAST tool). The results showed that humans are the main source of food for triatomines, indicating that human–vector contact is more frequent than previously thought. Although other groups of mammals, such as rodents, are also an available source of blood, birds (particularly chickens) might have a predominant role in the maintenance of triatomines in these areas. However, the diversity of sources of blood found might indicate a preference driven by triatomine species. Moreover, the presence of more than one source of blood in triatomines collected in the same place indicated that dispersal of vectors occurs regardless the availability of food. Dispersal capacity of triatomines needs to be evaluated to propose an effective strategy that limits human–vector contact and, in consequence, to decrease the risk of *T. cruzi* transmission.

## 1. Introduction

The identification of species present in blood meals is an important strategy to understand the implication of hematophagous vector species in disease transmission and their ecological interactions. Constant environmental changes, disease outbreaks and increases in vectors’ distribution pose challenges for understanding the transmission scenarios of vector-borne diseases. Important epidemiological traits of neglected tropical diseases (NTDs), such as the transmission of pathogens across multiple taxa, primary vector species, vertebrate species with a role in transmission and even social and economic conditions that foster transmission risk, have been assessed for important vectors such as *Culex* [1,2], *Phlebotomus* [3,4], *Aedes* [5] and triatomines [6,7].

Chagas disease (CD) is considered as one of the NTDs and it is caused by the infection of humans with *Trypanosoma cruzi*. The parasite is transmitted mainly through contact with the feces of infected triatomines. The primitive transmission of *T. cruzi* was restricted to the sylvatic transmission cycle (between triatomines and mammals without human participation); however, the gradual process of human settlement near the zoonotic transmission zone opened new niches for triatomines [8].

Understanding both cycles of transmission, enzootic and human, is fundamental to our understanding of the dynamics of *T. cruzi* and how its cycle can be targeted to control CD. This protozoan parasite has been reported in more than 100 mammalian species (seven orders) in diverse environments [9,10,11]. Although mammals can be infected by *T. cruzi*, not all mammals can be considered reservoirs, because it implies not only the infection but the infective potential and competence of the host [10]. Moreover, birds, amphibians and reptiles are refractory to *T. cruzi*; however, fowl may represent an important feeding source for triatomines and play an important role in transmission because they can increase the density of vector populations [12].

Even though it is difficult to delineate, there are three cycles involved in the transmission of *T. cruzi*: (i) the enzootic or sylvatic cycle includes wild mammals and triatomines living in nests and burrows. This cycle is a dynamic web with different routes of transmission that may or may not overlap [8,12,13]. In the human dwelling spaces, two cycles can be defined: (ii) the domestic cycle, when the transmission occurs indoors, involving, mainly, humans and domiciliated triatomines [8], and (iii) the peridomestic cycle that occurs between triatomines infesting manmade construction and structures around the house and domestic animals [14].

Persistence of these cycles depends on the availability and diversity of the vertebrate hosts [15]. To understand the dynamics of *T. cruzi* transmission and the epidemiology of CD, it is necessary to determine the diversity of vertebrates that are sources of blood for triatomines and its role in the different cycles.

In the central coastal and southern highland regions of Ecuador, five species of triatomines are considered as important vectors for *T. cruzi* transmission in domestic and peridomestic cycles: *Rhodnius ecuadoriensis*, *Panstrongylus chinai*, *Panstrongylus howardi*, *Panstrongylus rufotuberculatus* and *Triatoma carrioni* [16,17]. Moreover, *R. ecuadoriensis* represents a threat for vector control due to its broad geographic distribution and abundance in the sylvatic cycle [18,19]. In areas where triatomines were reported, *T. cruzi* has also been found infecting rodents (*Rattus rattus*, *Aegialomys xanthaeolus* and *Simosciurus nebouxii*), marsupials (*Didelphis marsupialis*) and bats (*Artibeus fraterculus*, *Myotis* sp. and *Glossophaga soricina*) [20,21,22]. However, the interactions between vectors and vertebrate hosts are not fully understood; in Ecuador, in particular, there is limited information regarding direct associations of triatomines with mammals [20,22], and the molecular identification of blood meals can accelerate our understanding of these interactions. Thus, the aim of this study is to evaluate the diversity of vertebrate hosts that serve as sources of blood and the infection rate with *T. cruzi* in triatomines collected in domestic, peridomestic and sylvatic transmission cycles in the central coastal and southern highland regions of Ecuador.

## 2. Materials and Methods

### 2.1. Triatomine Selection

**Figure 1:**
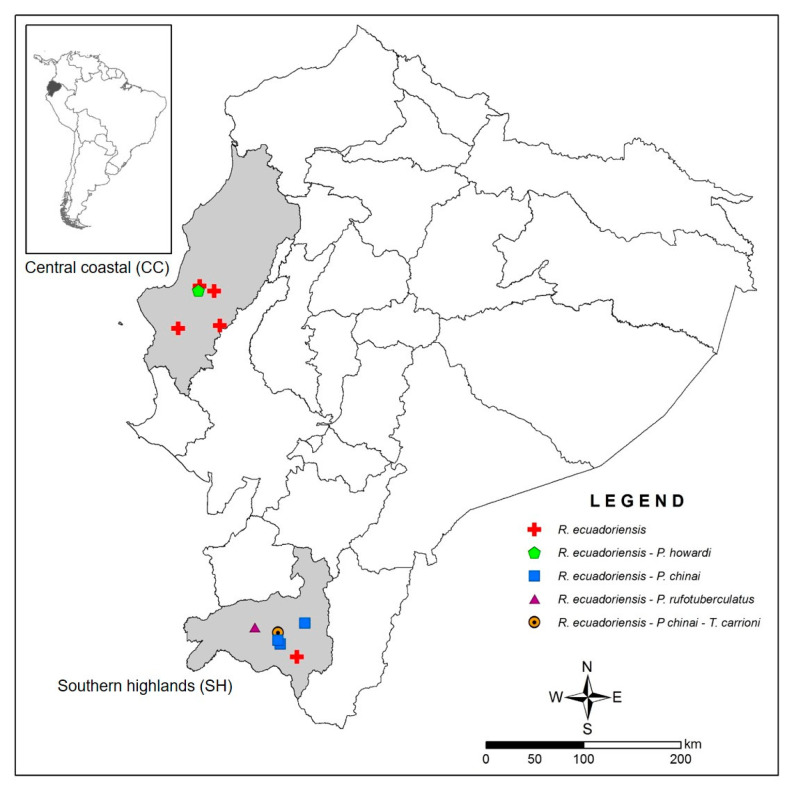
Geographic origin of 507 triatomines included in the analysis of blood meal sources. Each point indicates a locality and the different shapes and colors the triatomine species that were analyzed in each locality, as described in the legend. Central coastal corresponds to Manabí province and southern highlands to Loja province, from which 171 and 336 samples, respectively, were analyzed. Black lines represent the province limits.

Analyses were carried out in 507 selected samples of DNA purified from the intestinal content of five species of triatomines (60 *P. chinai*, 17 *P. howardi*, 1 *P. rufotuberculatus*, 427 *R. ecuadoriensis* and 2 *T. carrioni*) previously collected from 2006 to 2013 in the central coastal (CC) (*n* = 171) and southern highland (SH) (*n* = 336) regions of Ecuador (Figure 1). Intestinal contents were extracted from triatomines (235 adults and 272 nymphs). Most of the samples (*n* = 320, 63.12%) were extracted within 20 days post collection, and 36.88% were extracted from 21 to 40 days post collection. Variation in time from collection to extraction depended on the availability of a field laboratory (rapid containment kit, Germfree, Ormond Beach, FL, USA) to process the insects or the transfer and delayed process in the laboratory at CISeAL. All intestinal content samples were kept at −20 °C until DNA extraction. Triatomines were collected from three transmission cycles: (a) In the intradomicile (indoors), triatomines were collected in any structure inside the house, such as rooms (e.g., wall, bed, between clothes), piles of material (accumulation of tiles or bricks), animal shelters (bird or rat/mice nests). (b) The peridomicile cycle was considered the space around the house (usually delimited by a fence). Triatomines in the peridomicile were collected in piles of material (e.g., bricks, wood) or animal shelters of domestic and non-domestic animals (e.g., chicken, pigeon and rat/mice nests, dog and cat beds). (c) The sylvatic cycle (outside household limits) was mainly associated with animal shelters of non-domestic animals (e.g., squirrel, rat and bird nests), although it included areas where human activities can take place (e.g., forest patches with trails, areas for agriculture and harvesting, etc.).

### 2.2. DNA Extraction and Natural Infection with Trypanosomes

DNA was extracted from the homogenized intestinal content with the DNAzol reagent for the isolation of genomic DNA (Invitrogen^TM^, Waltham, MA, USA) in a DNA extraction hood that was sterilized with UV light before and after each use. All DNA samples were stored at −20 °C until use. Infection with *T. cruzi* was assessed by amplification of the variable domain of the minicircle of kDNA by the presence of a 330-bp product [23,24]. A negative sample (without DNA) and a positive control with DNA of *T. cruzi* were used in each PCR assay. Amplification products were detected in a 2% agarose gel stained with 1X SYBR Safe (Thermo Fisher Scientific, Waltham, MA, USA).

### 2.3. Comparison of Primer Sets for Detection of Blood Meals

Three different primer sets that amplified mitochondrial gene fragments were assayed with serial dilution of DNA from human blood (10 ng, 1 ng, 100 pg, 10 pg, 1 pg, 100 fg) to determine the most adequate for vertebrate host identification in DNA from stored intestinal content. Two sets of primers amplified a fragment of the *cytb* gene: FACytb-F (5′-CCC CTC AGA ATG ATA TTT GTC CTC A-3′) and FACytb-R (5′-CCA TCC AAC ATC TCA GCA TGA TGA AA-3′) [25]; and L14841 (5′-AAA AAG CTT CCA TCC AAC ATC TCA GCA TGA-3′) and H15149 (5′-AAA CTG CAG CCC CTC AGA ATG ATA TTT GTC-3′) [26]. Another set of primers amplified a fragment of the *12S*-*rNA* gene: L1091 (5’-AAA AAG CTT CAA ACT GGG ATT AGA TAC CCC-3′) and H1478 (5′-TGA CTG CAG AGG GTG ACG GGC GGT GTG T-3′) [26]. Amplification conditions are summarized in Table S1.

### 2.4. Sensitivity of the Different Primer Sets

The best performance of primer sensitivity using human DNA serial dilutions was achieved for the primers FACytb, where the amplification of a 355-pb fragment of *cytb* was obtained using up to 1 pg of DNA. For the two other primer sets (L14841-H15149 and L1091-H1478), the expected bands were obtained using up to 10 pg of DNA.

### 2.5. Amplification and Identification of the Sources of Blood Meals

Intestinal content DNA samples were amplified with the primer set FACytb, following the amplification conditions described in Table S1. Preparation of the mix was carried out in a pre-PCR hood sterilized with UV light before and after each use. A negative sample (without DNA) and a positive control with human DNA were used in each PCR assay. The expected 355-bp fragment was visualized in 2% agarose gels after SYBR Safe staining. Amplification products were sent for sequencing to Macrogen, Inc. (Seoul, Korea). Forward and reversed sequences were aligned with Mega V6.0 (https://www.megasoftware.net/manual.pdf). Consensus sequences were compared with DNA nucleotide sequences in NCBI BLAST using the megablast query. Matches were based on results achieving ≥95% maximum identity and cover. Statistical analysis was carried out with Chi^2^ and Fisher test to compare blood sources, transmission cycles and geographical regions.

### 2.6. Analyses of Trophic Networks of Triatomines and Their Blood Meals

To visualize the associations between the triatomines and the vertebrates which they feed on, we conducted trophic network analyses. We scored a matrix of interactions by counting the number of individuals of each species of triatomine (*n* = 5) that fed on a respective vertebrate species (*n* = 16). This matrix was analyzed with the functions *plotweb* and *visweb* using the library *bipartite* [27] in R version 3.6.2 [28].

## 3. Results

### 3.1. Vertebrate Diversity as Source of Blood for Triatomines

Of the 507 samples analyzed (Table S2), 82.05% of the samples (*n* = 416) were identified to blood meal species due to the sequences that matched with the ones in Genbank with an identity ≥ 95%. For 10.4% (*n* = 53) of the samples, PCR products were not obtained, and for 1.4% (*n* = 7), non-legible chromatograms or the presence of multiple peaks were present. For 6.1% (*n* = 31) of the samples, the obtained sequences matched with a reported species in GenBank, but the identity was <95%; among these last samples, we found birds (*Gallus gallus*, *Gallus lafayetii*), primates (*Homo sapiens*), rodents (*Rhipidomys leucodactylus*, *Simosciurus stramineus*, *Proechimys semispinosus*, *Rattus satarae* and *Rattus rattus*) and three species of insects that do not occur in the study area (*Rhodnius pictipes*, *Kleidocerys resedae* and *Tiniocellus spinipes*). None of the sequences with identity < 95% were included in the analysis.

**Figure 2:**
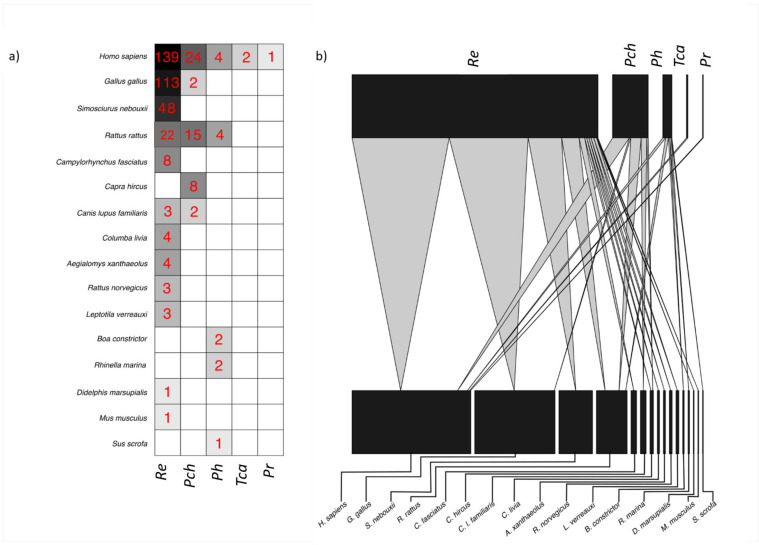
Trophic networks of the five species of triatomines (*R. ecuadoriensis*—*Re*, *P. chinai*—*Pch*, *P. howardi*—*Ph*, *P. rufotuberculatus*—*Pr* and *T. carrion*—*Tca*) and their food sources (vertebrates’ blood). (**a**) Grid representation of the trophic network; numbers in red indicate the number of triatomine specimens per species with a given blood meal species, and the intensity of the background color is proportional to these numbers (higher the frequency, darker the background); (**b**) bipartite graph of the trophic network.

For the 416 triatomines where blood meals were identified, sixteen vertebrate species were observed (Table 1). Trophic patterns of the five studied vector species are summarized in Figure 2. The class Mammalia represented 67.8% (*n* = 282) of the analyzed samples, with ten species, including five species of rodents (*Aegialomys xanthaeolus*, *Mus musculus*, *Rattus norvegicus*, *R. rattus*, *S. nebouxii* (previously known as *Sciurus stramineus*)), one carnivore (*Canis lupus familiaris*), two artiodactyla (*Capra hircus*, *Sus scrofa*), one didelphimorphia (*Didelphis marsupialis*) and one primate (*H. sapiens*). Four species of the class Aves were identified (*G. gallus*, *Campylorhynchus fasciatus*, *Columba livia* and *Leptotila verreauxi decolor*) and represented 31.2% (*n* = 130) of the analyzed samples. One species of the class Reptilia (*Boa constrictor imperator*) and class Amphibia (*Rhinella marina*) (previously known as *Bufo marinus*) was identified in <1% (*n* = 4) of the samples. Overall, the most abundant vertebrate hosts identified as sources of blood for triatomines were humans (*H. sapiens*) (*n* = 170, 40.9%), chickens (*G. gallus*) (*n* = 115, 27.6%) and rodents (*S. nebouxii* and *R. rattus*) (*n* = 89, 21.4%).

All five triatomine species analyzed were found to have ingested human blood. Only two triatomine species—*R. ecuadoriensis* and *P. chinai*—ingested chicken blood and *R. ecuadoriensis* was the only species of triatomine that had ingested blood of the four recorded bird species (*G. gallus*, *C. fasciatus*, *C. livia* and *L. verreauxi*). In *P. howardi*, blood meal from the amphibian (*R. marina*) and the reptile (*B. c. imperator*) was found. Only human blood meal was identified in the few specimens of *T. carrioni* and *P. rufotuberculatus* (Figure 2).

### 3.2. Vertebrate Blood Meal Source per Geographic Area, Triatomine Species, Developmental Stage and Transmission Cycle

**Figure 3:**
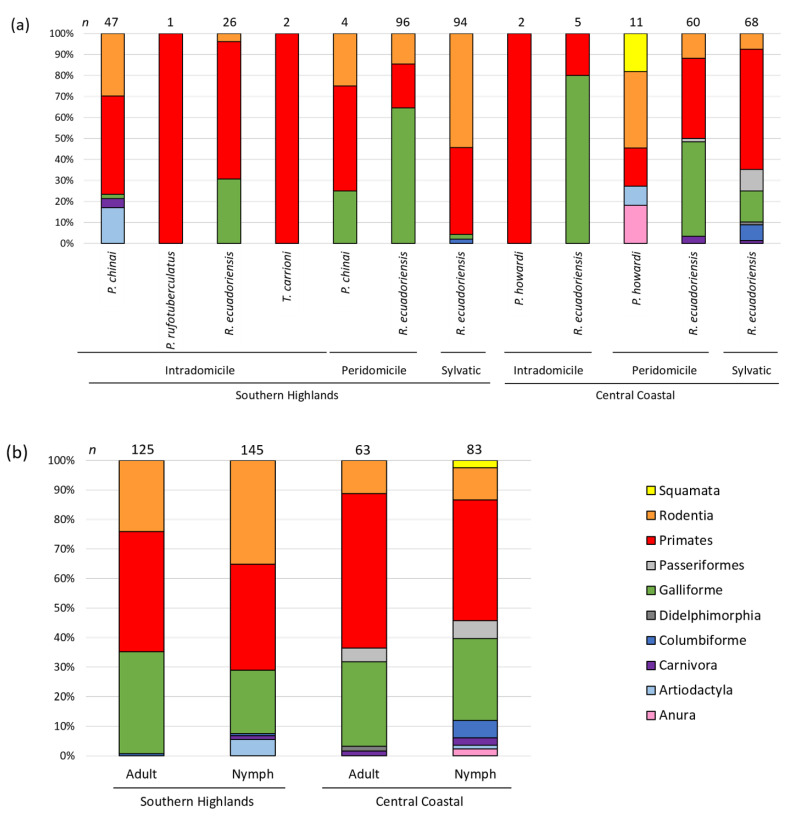
Distribution of vertebrate orders found as source of blood of triatomines per (**a**) region, transmission cycle and vector species; (**b**) region and vector developmental stage.

The blood meal distribution per species in the two regions is presented in Figure 3a. Overall, blood meal vertebrate diversity appeared higher in CC (14 blood meal sources) than in SH (eight blood meal sources), but this difference was not significant (Fisher exact test, *p* > 0.05). A similar observation was made for *R. ecuadoriensis,* the only studied species present in both regions; of a total of 12 blood meal hosts, 11 were identified in CC and 6 in SH (Fisher exact test, *p* > 0.05). For *P. howardi*, a species only found in CC, five sources of blood were identified, including three mammals (Human, *R. rattus* and *S. scrofa*), one species of amphibian (*R. marina*) and one species of reptile (*B. constrictor imperator*); these last two are non-typical blood sources for triatomines.

In the SH region, human was the most abundant source of blood for triatomines, particularly for the specimens collected in the intradomicile (*P. chinai, P. rufotuberculatus*, *R. ecuadoriensis* and *T. carrioni*), but also it was frequently found in the species that infested the peridomestic and sylvatic cycle (*P. chinai,* and *R. ecuadoriensis*). In the CC region, human blood meal was found in all three transmission cycles but was particularly abundant in *R. ecuadoriensis* from the peridomicile and sylvatic cycles (Figure 3a). For *R. ecuadoriensis*, chicken (*G. gallus*) and rodents (*S. nebouxii*, *R. rattus*) are other important sources of blood. For *P. chinai*, rodents (*R. rattus*) and, to a lesser extent, ungulates (Artiodacyla—*C. hircus*), carnivores (*C. lupus familiaris*) and birds (*G. gallus*) were also detected as important sources of blood (Figure 3a).

In both regions, sources of blood meal were identified in adults and nymphs. Overall, no significant distribution of the main blood meals (human, chicken (*G. gallus*) and rodent (*R. rattus* and *S. nebouxii*)) was observed between adults and nymphs (Fisher exact test, *p* = 0.11; Figure 3b), even if a higher diversity of hosts was observed in nymphs (15 blood meal sources) than in adults (10 blood meal sources), but this was not significant (Fisher exact test, *p* > 0.05) (Figure 3b).

Blood meal sources were identified in triatomines collected in the intradomicile, peridomicile and sylvatic cycles. In total, considering three categories of blood meals, the two most frequent ones (human and *G. gallus*) and a third category grouping all the others, the observed distribution was significantly different between the transmission cycles (Chi^2^ = 96.14, df = 4, *p* < 0.0001). Humans tend to be overrepresented in blood meals of triatomines captured in the intradomicile cycle (the observed frequency was 32.7% greater than the expected) and, to a lesser extent, in those captured in the sylvatic cycle, where the observed frequency was 17.8% greater than expected. In the peridomicile, *G. gallus* were overrepresented in the blood meals of triatomines and the observed frequency was 90.4% greater than expected.

**Figure 4:**
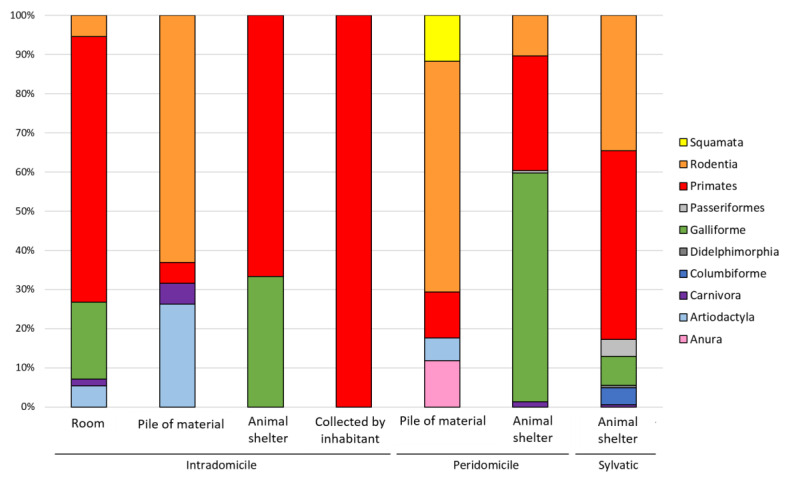
Distribution of vertebrate orders found as sources of blood of triatomines according to the microenvironment of collection.

To refine our analysis, we examined the sources of blood meals of the triatomines according to the microenvironment in which they were captured, as presented in Figure 4. In the intradomicile, triatomines were mostly found in rooms (*n* = 56, 67.4%), particularly in bedrooms (in the bed, between clothes and on the wall). As expected, in this transmission cycle, the main source of blood was human (54.2%), although species of other orders were found, such as Galliformes (*G. gallus*), Rodentia (*R. rattus*), Artiodactyla (*C. hircus*) and Carnivora (*C. lupus familiaris*). Triatomines were also found in piles of material (bricks or wood) stored inside the house. Most of the triatomines found in this microenvironment had Rodentia blood (*R. rattus*); however, blood from other sources, such as human, goat (Artiodactyla) and dog (Carnivora), was also found. Another microenvironment present in the intradomicile (although less frequent) was animal shelters (chicken and rodent nests). Triatomines collected in this microenvironment presented blood from chicken and human. In the few triatomines collected indoors by the inhabitants (triatomines attracted by light), only human blood was detected (Figure 4).

In the peridomicile, most of the studied triatomines were collected in animal shelters (mostly chicken nests and some others in rodent nests). In these microenvironments, chicken or rodent blood meals were the most frequent, but human blood meals were also detected. Triatomines collected in piles of material (mainly bricks and woods) presented different blood meal sources such as *R. rattus*, humans, *S. scrofa*, *B. c. constrictor* and *R. marina* (Figure 4).

In the sylvatic cycle, all studied triatomines were collected in animal shelters, mainly squirrel nests (87.0%). A total of 11 different blood meal sources were identified, with human and the squirrel, *S. nebouxii*, being the most abundant. A small proportion of triatomines had blood meals of domestic and non-domestic species of the order Rodentia (*R. rattus*, *A. xanthaeolus* and *M. musculus*), Carnivora (*C. lupus familiaris*), Didelphimorphia (*D. marsupialis*), Galliformes (*G. gallus*), Columbiformes (*L. v. decolor*, *C. livia*) and Passeriformes (*C. fasciatus*) (Figure 4).

### 3.3. T. cruzi Infection and Blood Meal Diversity in the Different Transmission Cycles

**Figure 5:**
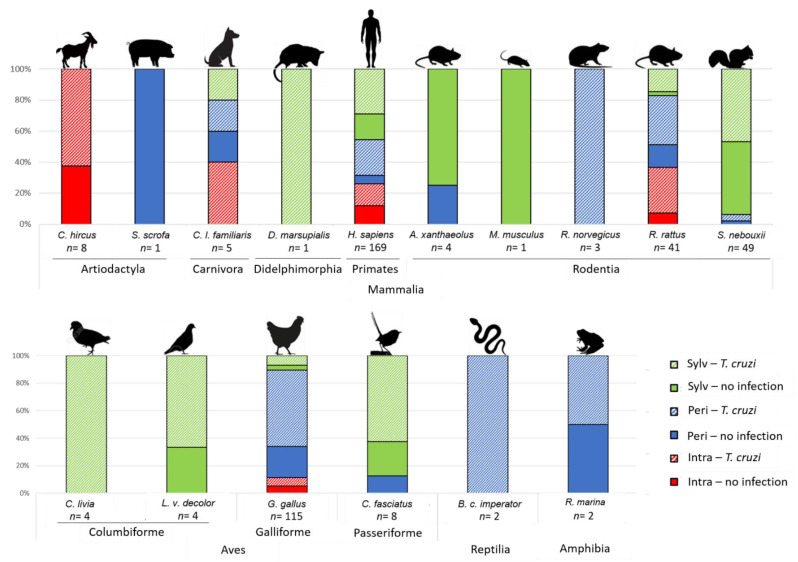
Proportion of the infected and non-infected triatomes with *T. cruzi* among those collected in different transmission cycles with a given blood meal (species of vertebrate). Intra = intradomicile, Peri = peridomicile and Sylv = sylvatic cycle.

Of the 416 triatomines with identified blood meals, 65.9% were infected with *T. cruzi*. Table 1 and Figure 5 summarize the infection rates observed according to each blood host species and transmission cycle. The infection rate was similar in triatomines fed with mammal and bird blood (64.2% and 69.2%, respectively, Chi^2^ = 0.04, *p* > 0.05). Similarly, adults presented a *T. cruzi* infection rate (67.6%) not different from that of nymphs (64.5%) (Chi^2^ = 0.31, *p* > 0.05).

Triatomine infection rates with *T. cruzi* collected in the domicile and sylvatic cycle reach around 60% (Chi^2^ = 0.02, *p* > 0.05); these values were significantly lower than those of peridomestic cycle (Chi^2^ values of 4.31 and 5.43, respectively, *p* < 0.05). In the domestic cycle, the infection rate of triatomines with mammal blood meals (61.4%) or bird blood meals (53.8%) were not significantly different (Fisher exact test, *p* > 0.05). Additionally, the infection rate in triatomines with rat blood (80.0%) tends to be higher than those with human blood (53.3%), but this difference is not statistically significant (Fisher exact test, *p* > 0.05).

In the peridomestic cycle, eleven species of vertebrates were identified as sources of blood meal (seven mammal, two bird, one reptile and one amphibian species); no significant differences in infection rates were observed between the principal sources of blood meals, chickens (71.1%), rats (68.4%) and humans (83%) (Fisher exact test, *p* > 0.05). In this cycle, *T. cruzi* was detected in one of the two amphibian blood meals and in both samples with reptilian blood meals (Table 1, Figure 5).

In the sylvatic cycle, species of vertebrate hosts (7 species of mammals, 4 species of birds) were found as sources of blood for triatomines, with mammals being the most frequent source, with a 58.8% *T. cruzi* infection rate. The mammal group included domestic and synanthropic animals, such as dogs, opossums, mice and rats, and sylvatic ones, such as yellowish rice rats and squirrels. Surprisingly, human blood was found in 48.4% (*n* = 78) of the sylvatic triatomines, with a *T. cruzi* infection rate as high as 62.8%. Overall, the *T. cruzi* infection rate in birds (domestic and wild) reached 73.1% (Table 1, Figure 5). A non-statistical difference was found when we compared human, squirrel and bird blood meals (Fisher exact test, *p* > 0.05).

## 4. Discussion

### 4.1. Epidemiological Importance of the Identification of the Vertebrate Sources of Blood for Triatomines

Triatomines feed on a wide diversity of vertebrate species, and the detection of these hosts in a given area is essential to understand the interactions between triatomines and vertebrate hosts and to identify important reservoir hosts of CD. In Ecuador, a great deal of information has been obtained about the diversity and ecological conditions of triatomines and *T. cruzi* [16,17,29,30], which is crucial to define target species of triatomines for their control. Although insecticide spaying was successful to control *T. dimidiata* populations, limited effectiveness was observed when applied to the control of other domiciliated species [31]. Likewise, the continuous reinfestation of houses constitutes a challenge for effective control measures to avoid human–vector contact.

To find alternative and comprehensive measures to fight against CD transmission, information about the contact between the triatomine vectors and the vertebrate hosts is essential. The identification of the sources of blood meal, and, at the same time, the detection of *T. cruzi* infections, contributes to: (1) defining the rate of human–vector contact, which informs us about the risk of CD transmission, (2) determining the most frequent sources of blood, other than humans, that allow vector populations to thrive, (3) the infection rate of the vectors that is considered a second risk index of CD transmission, (4) the potential mammal reservoirs that could be targeted for CD control, (5) the knowledge of the dynamics of transmission through the cross-analysis of the place of capture of the vectors, the source of the blood meal of each vector and the proximity of the potential hosts, and (6) the vector species composition and their ability to transmit the parasite.

The present study answered some of the questions listed above. Among others, this study described the great diversity of vertebrate hosts that are sources of blood for the main vector species in Ecuador, the high *T. cruzi* infection rate of triatomines, even when they are captured in the nests of fowls (laying hens or other birds), which are refractory to *T. cruzi* infection, and the high frequency of human blood meals in vectors captured indoors but even in those captured outside the home.

### 4.2. Diversity of Mammal Hosts and Its Implication in T. cruzi Transmission

Mammal species are recognized as frequent blood sources and the most important because these species can be infected and transmit *T. cruzi*. In this study, mammals contributed 67.8% of the identified feeding sources; of these, human, black rat (*R. rattus*) and squirrel (*S. nebouxii*) represented together 91.8%. The two first species were found in triatomines collected in the domestic, peridomestic and sylvatic cycles, while squirrels contributed above all to blood sources in the sylvatic cycle.

Human blood has been previously found in the three principal triatomine genera implicated in *T. cruzi* transmission (*Panstrongylus, Rhodnius* and *Triatoma*) [32,33,34,35]; however, the high proportion of human blood meals in sylvatic triatomines in both regions, while they have different ecological conditions, was unexpected. Contact of humans with sylvatic triatomines has been proposed to be related to outdoor activities (e.g., camping, harvesting) or to the movement of triatomines from sylvatic to domestic settings and vice versa [36,37,38]. In the present context, such human activities and possible human–vector contact outside the home as well as triatomine dispersal need to be further studied.

Endemic and wild species of rodents are important blood sources for triatomines, particularly in the sylvatic cycle [36,39,40,41]. In this study, apart from humans, triatomines mostly fed from squirrels (*S. nebouxii*) and a few other rodent species. In previous studies, sylvatic triatomines have been searched in burrows, tree holes, under trunks and rocks, but they were mainly collected in nests built in trees, particularly in squirrel nests [18,19,42]. Although in CC, the blood meals of sylvatic triatomines were more diverse and squirrels did not seem to be an important source of blood, in SH, these results support the importance of *S. nebouxii* in the maintenance of triatomine populations in forest patches around rural communities [43].

Synanthropic rodents such as rats (*R. rattus*) have been found to be key sources of blood for triatomines in some areas such as Bolivia, Mexico (where rats play a major role as feeding source and should be the main reservoir of peridomicile colonies of *T. longipennis*) or Guatemala (where rodent control was applied as part of a strategy for an integrated vector management) [36,44,45,46]. However, in Panama, Argentina, Venezuela and Colombia, rats are not an important source of blood for triatomines [32,33,34,35]. In our studied areas, the results showed a low frequency of feeding from rat blood (15.6% of mammal blood meals), although rats have the capacity to circulate between sylvatic and domestic cycles. The contact of rats with triatomines might be less frequent than expected, even if they are widely distributed; thus, in Ecuador, their role in *T. cruzi* transmission would be more dependent on local ecological characteristics.

The opossum is considered a natural host species for the parasite and it is an important reservoir in some areas in Colombia and Brazil [35,46,47,48]. However, based on this and a previous study [21], opossums seem to have a limited role in triatomine maintenance and *T. cruzi* transmission in Ecuador, although this is an abundant species.

The role of dogs also differs by region. In general, in Chagas-disease-endemic areas, there is a high frequency of dogs infected with *T. cruzi* and their presence in dwellings has been associated with higher risk of human infection [47,49]. In Colombia, dogs have been found as an important source of blood for *T. maculata* [35]. In other areas, such as the USA (Texas and Louisiana) and Brazil, the *T. cruzi* seroprevalence of dogs ranged from 7 to 76% [50,51,52,53]. In Northern Argentina, infected dogs constitute a risk factor and have been used as sentinels for *T. cruzi* domestic transmission [54,55]. However, in other areas, dogs might not have such an important role, although they have been found to be a less frequent source of blood [56,57]. In areas of Ecuador in which Chagas disease is endemic, dogs are present in the majority of domiciles [16,17]; nevertheless, the results of this study indicate that dogs are not a major source of food for triatomines, despite their availability.

Other mammals, such as the mouse (*M. musculus*), the brown rat (*R. norvegicus*), goats (*C. hircus*) and pigs (*S. scrofa*), might be occasional sources of food and could play a limited role in *T. cruzi* transmission in Ecuador.

### 4.3. Birds and Their Role in Triatomine Population Maintenance

Birds do not have a direct role in *T. cruzi* transmission because they are refractory to the parasites; they are able to destroy the trypomastigote form through the action of their immune system. However, they are a source of blood that plays an important role in the survival and maintenance of triatomine populations [12], particularly in rural areas where raising chickens is frequent. Experimental studies in *T. infestans* have demonstrated that this vector species prefers feeding from dogs than from chickens [58]. In Ecuador, having chickens was found to be a major determinant of triatomine infestation and *R. ecuadoriensis* is the vector species that showed a preference for this microenvironment [16,17,59]. Most chickens are kept free-ranging, except for laying hens, whose nests are located outside against the house walls so to be easily monitored by the inhabitants [60]. Large colonies of triatomines have been found in these nests. Although chickens are the expected source of blood for triatomines collected in chicken nests (63.6%), we also found human (31.8%) and rodent blood meals (4.6%). Moreover, triatomines that contained chicken blood were found infected by *T. cruzi*. These last findings show that triatomines, adults and nymphs, move from one microenvironment to another; thus, the dispersal of triatomines between the intradomicile, peridomicile and sylvatic cycles might play an important role in transmission. According to the present results, other birds, such as doves, pigeons and others, might contribute to the maintenance of triatomine populations in the sylvatic cycle.

### 4.4. Triatomine Species and Blood Meal Preference

*Rhodnius ecuadoriensis* is the most abundant and widely distributed species found in the two studied regions; moreover, it colonizes all three transmission cycles [16,17]. In both regions, given that blood meals taken from humans are frequent, regardless of the transmission cycle where the bugs were collected (27.6% in peridomicile to 58.1% in intradomicile), it can be argued that this species has remarkable anthropophilic behavior. The most conclusive observations are those made in specimens collected in the sylvatic cycle: in CC, although a variety of hosts is observed (10 species of vertebrates counted), the human food represents 57.3%; in SH, they are slightly less represented (41.5%) since this species is often found in the nests of squirrels and meals from them reached 46.8%. Among these insects, around half are adults who have thus moved to rely on humans for food, while the nymphs feed from their congeners engorged with human blood, as discussed in the next section.

In *P. chinai*, whose presence is well established in domestic and peridomestic cycles in SH only [17], of the 51 specimens studied, around half have also fed on humans. *R. rattus* seems to be the second most preferred species by *P. chinai*, when collected in intradomicile, while this is not the case for *R. ecuadoriensis*, which shares the same region and same villages. Detection of other sources of blood, such as the dog, goat, rat and chicken, however, shows flexibility in food choices, a characteristic conducive to the adaptation of species to new environments.

What is striking for *P. howardi* compared to other species is the diversity of diet observed in specimens collected in the peridomicile, which included humans, rats, reptiles and amphibians; no chicken blood meals were detected, even though chickens are abundant in the peridomicile, but the sample size was small.

All these results might indicate that although triatomines have a diverse diet, some species showed blood source preferences that can have, as in the case of *R. ecuadoriensis*, important implications in *T. cruzi* transmission to humans.

### 4.5. Triatomine Dispersal and Access to Vertebrate Hosts

To understand the dynamics of transmission in an endemic area, knowing the movements of triatomines is very informative. In this context, the current cross-study that considered the places where each triatomine was captured with its blood meal offers valuable information, even if the study was limited to a single meal per triatomine. Indeed, several triatomines had multiple meals (1.4%) that were reflected by multiple peaks in the chromatograms, showing an overlap of *cytb* sequences. Although the resolution of the multiple meals by molecular cloning was not applied, detecting multiple sources of blood could facilitate the understanding of the ecological interactions of triatomines [6,61].

In this study, triatomines collected in chicken nests in the peridomicile and squirrel nests in the sylvatic cycle presented unexpectedly a high diversity of blood sources. Furthermore, triatomines collected in all transmission cycles, including sylvatic, showed the presence of human and chicken blood, showing that these triatomines had fed elsewhere before arriving in these places. The dispersal capability of triatomines has been previously reported. In Argentina, the dispersion distance range of adult *T. infestans* has been recorded from 0.1 up to 2 km [62,63]. In Mexico, an elevated dispersal between domestic and sylvatic areas was suggested for *T. dimidiata* [64]. In the Andean valley in Bolivia, the evaluation of dispersal distances of *T. infestans* by the capture–mark–recapture method in the field demonstrated significant movement in adults of up to 168 m and in nymphs of 34 m [65].

Unexpected blood meals of nymphs that differ from the available host in the place of capture are not easy to explain. Nymphs have a limited capacity for dispersion due to the lack of wings, and in some cases, it is not possible to assume that the nymph located its meal by walking, so other strategies such as cleptohematophagy or cannibalism, which have been previously reported under experimental conditions for some triatomine species, can be proposed [66,67,68]. Alternatively, nymphs could be passively dispersed by transportation by animals or humans, as has been reported for *Triatoma brasiliensis*, *Rhodnius prolixus* and *Triatoma pseudomaculata* [69,70,71].

Host accessibility is a major factor that shapes the patterns of the sources of blood for triatomine, and it varies according to the studied area [72]. In Panama, anthropogenic landscape disturbance changed the host community structure and increased *R. prolixus* infection with *T. cruzi* [15]. Conversely, in Argentina, domestic animals and their availability throughout the year affect triatomine´s host-feeding choices and even human–vector contact rates [73]. In Ecuador, triatomines have accessibility to domestic animals such as dogs and chickens [16]. Although chickens are an important blood source for *R. ecuadoriensis*, humans constitute the most important blood source for all five vector species analyzed. In Ecuador, the dispersal and dynamics of triatomines, particularly *R. ecuadoriensis*, need to be further evaluated to determine the factors that influence the human–vector contact in the domestic cycle and the possible scenario that can explain human blood meals in specimens collected in the sylvatic cycle.

## 5. Conclusions

Humans are the main source of blood for the current sample of triatomines collected in Ecuador, indicating that human–vector contact is more frequent than previously thought. This result, together with the high infection rates with *T. cruzi*, indicates a high risk of transmission of the parasite in the central coastal and southern highland regions of Ecuador. Moreover, the high frequency of chicken blood in peridomestic triatomines indicates that this species is fundamental in the maintenance and thriving of triatomine populations in household environments. The triatomine *R. ecuadoriensis*, which is the most abundant species in both studied regions and widely distributed in the domestic and sylvatic cycles, appeared to prefer human and chicken blood, having a principal epidemiological role. Moreover, other species that colonized peridomestic environments, such as *P. chinai* and *P. howardi*, might prefer human and rodent sources of blood but not chicken, taking second place in the risk of transmission. Both adults and nymphs, who are similarly infected with *T. cruzi*, come into contact with humans; even nymphs, which have limited dispersal because they cannot fly, can acquire human blood by other mechanisms such as cleptohaematophagy on their congeners. The dispersion profile of triatomines is obviously very complex. They are influenced by the availability of vertebrates, their own trophic preferences, but also the environmental disturbances that lead them to move and adapt to new sources of blood. The current results show that adults move from the outside to the inside of the house and vice versa, and the same applies for nymphs in some situations, such as that of the nests of laying hens located against the outside walls of houses. Both adults and nymphs therefore represent a risk of transmission. In this context, additional studies will provide a better understanding of the dispersal patterns of vector species, but it is important to understand from now on that these vectors move extremely easily between the sylvatic cycle and the human habitat and that they are able to adopt man as a food source, increasing the risk of *T. cruzi* transmission.

## Figures and Tables

**Table 1:** Diversity of feeding host species and *T. cruzi* infection identified in triatomines collected in intradomicile, peridomicile and sylvatic cycles in the central coastal and southern highland regions in Ecuador.

Vertebrate Blood Source		I *n* (Tc %)	P *n* (Tc %)	S *n* (Tc %)	Total *n* (Tc %)
Species	Common Name				
Mammalia					
Artiodactyla					
*Capra hircus*	Goat	8 (62.5)	-	-	8 (62.5)
*Sus scrofa*	Pig	-	1 (0.0)	-	1 (0.0)
Carnivora					
*Canis lupus familiaris*	Dog	2 (100.0)	2 (50)	1 (100.0)	5 (80.0)
Didelphimorphia					
*Didelphis marsupialis*	Opossum	-	-	1 (100.0)	1 (100.0)
Primate					
*Homo sapiens*	Human	45 (53.3)	47 (83.0)	78 (62.8)	170 (65.9)
Rodentia					
*Aegialomys xanthaeolus*	Yellowish rice rat	-	1 (0.0)	3 (0.0)	4 (0.0)
*Mus musculus*	Mouse	-	-	1 (0.0)	1 (0.0)
*Rattus norvegicus*	Brown rat	-	3 (100.0)	-	3 (100.0)
*Rattus rattus*	Black rat	15 (80.0)	19 (68.4)	7 (85.7)	41 (75.6)
*Simosciurus nebouxii*	Guayaquil squirrel	-	3 (66.7)	45 (51.1)	48 (52.1)
Total mammalia		70 (61.4)	76 (76.3)	136 (58.8)	282 (64.2)
Aves					
Columbiforme					
*Columba livia*	Pigeon	-	-	4 (100.0)	4 (100.0)
*Leptotila verreauxi decolor*	White-tipped dove	-	-	3 (66.7)	3 (66.77)
Galliforme					
*Gallus gallus*	Chicken	13 (53.8)	90 (71.1)	12 (66.7)	115 (68.7)
Passeriforme					
*Campylorhyncus fasciatus*	Fasciated wren	-	1 (0.0)	7 (71.4)	8 (62.5)
Total aves		13 (53.8)	91 (70.3)	26 (73.1)	130 (69.2)
Reptilia					
Squamata					
*Boa constrictor imperator*	Boa	-	2 (100.0)	-	2 (100.0)
Total reptilian		-	2 (100.0)	-	2 (100.0)
Amphibia					
Anura					
*Rhinella marina*	Cane toad	-	2 (50.0)	-	2 (50.0)
Total amphibian		-	2 (50.0)	-	2 (50.0)
Triatomines					
TOTAL		83 (60.2)	171 (73.1)	162 (61.1)	416 (65.9)
Adults		32 (53.1)	88 (71.6)	68 (69.1)	188 (67.6)
Nymphs		51 (64.7)	83 (74.7)	94 (55.3)	228 (64.5)

I: intradomicile, P: peridomicile, S: sylvatic; Tc %: *T. cruzi* infection detected by kDNA PCR.

## Data Availability

The data presented in this study are available in the Supplementary Materials Tables S1 and S2.

## Supplementary Materials

The following are available online at https://www.mdpi.com/2076-0817/10/1/42/s1, Table S1 Thermal cycling parameters for PCR primers tested according to previous publications. Table S2 Geographical and vector origin of the samples analyzed. Includes information of transmission cycle, *T. cruzi* infection, and sequence obtained from the *cytb* analysis.
